# A Cucumber Mosaic Virus Based Expression System for the Production of Porcine Circovirus Specific Vaccines

**DOI:** 10.1371/journal.pone.0052688

**Published:** 2012-12-20

**Authors:** Ákos Gellért, Katalin Salánki, Kata Tombácz, Tamás Tuboly, Ervin Balázs

**Affiliations:** 1 Department of Applied Genomics, Agricultural Institute, Centre for Agricultural Research, Hungarian Academy of Sciences, H-2462 Martonvásár, Brunszvik, Hungary; 2 Agricultural Biotechnology Center, H-2100 Gödöllő, Szent-Györgyi Albert, Hungary; 3 Department of Microbiology and Infectious Diseases, Szent István University Faculty of Veterinary Science, Budapest, Hungary; Federal University of Pelotas, Brazil

## Abstract

Potential porcine circovirus type 2 (PCV2) capsid protein epitopes, suitable for expression on the surface of cucumber mosaic virus (CMV) particles were determined by a thorough analysis of the predicted PCV capsid protein structure. The *ab initio* protein structure prediction was carried out with fold recognition and threading methods. The putative PCV epitopes were selected on the basis of PCV virion models and integrated into the plant virus coat protein, after amino acid position 131. The recombinants were tested for infectivity and stability on different *Nicotiana* species and stable recombinant virus particles were purified. The particles were tested for their ability to bind to PCV induced porcine antibodies and used for specific antibody induction in mice and pigs. The results showed that PCV epitopes expressed on the CMV surface were recognized by the porcine antibodies and they were also able to induce PCV specific antibody response. Challenge experiment with PCV2 carried out in immunized pigs showed partial protection against the infection. Based on these results it was concluded that specific antiviral vaccine production for the given pathogen was feasible, offering an inexpensive way for the mass production of such vaccines.

## Introduction

Vaccines have been revolutionary for the prevention of infectious diseases especially in case of virus induced clinical conditions. Beside the continued development of the inactivated or live attenuated vaccines, major efforts are invested in subunit vaccines [1] for either mucosal or parenteral delivery to overcome shortages of the traditional types. Subunit vaccine refers to pathogen-derived antigens, sometimes limited to one or more immunogenic domains of a protein, which cannot cause disease but can, activate the host immune response system against the pathogen. A special area of subunit vaccine production is offered by the use of plants, either as transgenic plants or as natural media for the propagation of recombinant plant viruses expressing a desired gene of an animal or human pathogen. Such vaccines, also as edible ones, have been at the focus of research since the first report of transgenic tobacco plants expressing hepatitis B surface antigen (HBsAg) [2], proving that HBsAg can stimulate mucosal immune responses via the oral route [3]. Using similar approaches a number of important veterinary pathogens were targeted and the vaccines showed promising results after oral or parenteral application [4]–[7]. Plant derived antigens offer several advantages over traditional vaccines, including stability, increased safety, rapid and massive production, cost effectiveness and especially in case of plant seeds long term storage and long distance shipment at variable temperatures [8]–[11].

The pioneering work of Lomonossoff’s group on a versatile plant virus expression system based on the icosahedral cowpea mosaic virus (CPMV) gave a burst to the development of alternative expression systems from plant RNA viruses [12]–[14]. One of the first such experiments used CPMV after the crystal structure of the virus particle had been resolved, allowing for the precise insertion of the epitopes into the coat proteins [15]–[17]. The number of similar expression systems using plant viruses is constantly increasing [18]. Recently cucumber mosaic virus (CMV) was considered as a potential vector for expressing foreign epitopes [19], [20]. Furthermore, CMV is a promising candidate as an oral vaccine, since it has an extremely wide host range, and accumulates in substantial amount in different parts of the plants, like leaves, fruits, tubers and roots.

A target of plant virus based vaccine development is suggested in the present study, namely against porcine circovirus (PCV) infections. PCV is one of the smallest known animal viruses; it belongs to the *Circovirus* genus of the *Circoviridae* family. It is non-enveloped with a single-stranded circular DNA genome surrounded by a capsid built of the only structural protein of the virus (the capsid protein) that is also the main target of antiviral immune response [21]–[23]. Two species of PCV have been identified so far, PCV1 [24] originally isolated as a cell line contaminant and PCV2 that is strongly immune suppressive and is responsible for a number of clinico-pathological conditions, referred to as PCV associated diseases (PCVD) [25]. PCV2 is present worldwide causing major economic losses in the pig industry. The control of the infection and PCVDs is crucial and traditional inactivated or subunit vaccines using baculovirus expression systems had been developed and commercialized. The efficacy of the available vaccines, based on the average daily weight gain and on the mortality rate in vaccinated herds is limited, but with beneficial effect on both [26].

**Figure 1:**
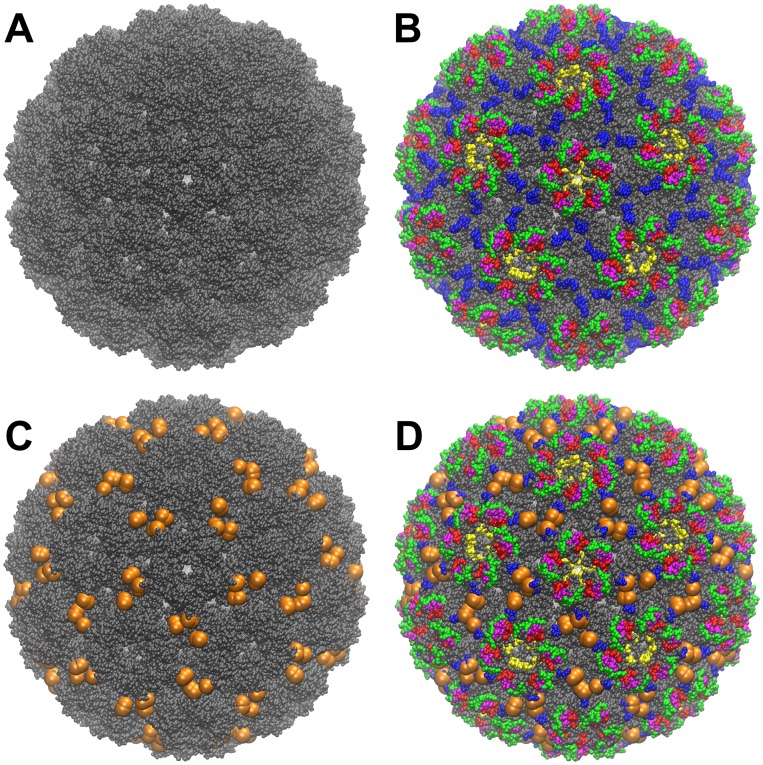
Molecular surface representations of the CMV virion. The whole molecular surface is colored in gray (A). External coat protein loops are colored as follows (B): loop 1 (76–83 aa) is green, loop 2 (113–118 aa) is red, loop 3 (129–136 aa) is blue, loop 4 (156–163 aa) is magenta and loop 5 (193–199 aa) is yellow. The PCV2 epitope insertion points are indicated in panel C. Ser 131 and 132 are represented as orange beads. Panel D is the superposition of B on C.

The detailed studies of the functions of important loops on the coat protein subunits present on the surface of cucumoviruses [27]–[31] initiated the current study to produce CMV expressing an epitope of PCV2 (Fig. 1) that is important in the induction of protective immunity against PCVDs.

## Materials and Methods

### Ethics Statement

The animal experiments were carried out in accordance with the Guidelines for Animal Experiments of the Szent István University and with EU Directive 2010/63/EU. The protocol was approved by the Committee on the Ethics of Animal Experiments of the Szent István University and the Central Agricultural Office (Directorate of Animal Health and Animal Welfare, Budapest, Hungary, Permit Number: 22.1/1020/3/2010). The animals were carefully monitored for any sign of distress, and all efforts were made to minimize animal suffering. Both mice and pigs remained symptomless throughout the study.

### Prediction of the PCV2 Capsid Protein Structure and Molecular Graphics

The PCV2 capsid protein (CP) structure was generated with I-TASSER [32], [33]. The model was built using the PCV2 CP sequence (NCBI/GenBank accession number: AAC35310). The following experimentally determined templates were used to thread the PCV2 CP structure: PDB ID codes: 2EIG (*Lotus tetragonolobus* seed lectin), 1V6I (Peanut lectin), 1C8N (Tobacco necrosis virus CP), 1NT0 (CUB1-EGF-CUB2 region of mannose-binding protein), 1NG0 (Cocksfoot mottle virus CP), 1JOD (pituitary adenylatecyclase-activating polypeptide), 1E4B (L-fuculose-1-phosphate aldolase). A PCV2 CP pentamer was created with Symmdock [34] for graphical epitope visualization. The model structures were refined with the Schrödinger molecular modeling software package (Schrödinger Suite) to eliminate the steric conflicts between the protein side chain atoms. Molecular graphics were created with VMD version 1.9 [35]. The virion models were created with the Oligomer Generator application of VIPERdb (available at http://viperdb.scripps.edu/oligomer_multi.php). Prior to virion model generations the asymmetric units were constructed with Schrödinger Suite using the coordinates of subunit A, B and C of CMV CP.

### Plasmid Constructs

R-CMV and the infectious transcripts (pR1, pR2, pR3) derived from this strain have been described previously [36]. The recombinant RNA 3 clones were generated by polymerase chain reaction (PCR) based mutagenesis of pR3. Oligonucleotide primers to insert the PCV derived regions were designed with the SilMut software package [37]. Oligonucleotides for pR3/131-PCV126-145 contained *Sna*BI restriction endonuclease (RE) site, and *Bam*HI for pR3/131-PCV224-233. The oligonucleotides were as follows:

R3/131PCV126-145 forward: 5′-GGG **TAC GTA CGA CCC ATA TGT AAA CTA CTC A** *TC CGA TCT TTC CGT CG*-3′, R3/131PCV126-145 reverse: 5′-GGG **TAC GTA AGT GCT GTT GCT TTA GTG ACA AAG TTA TCG TCT CC** *T GAA AGG TA* *CTT TCC GAA CTG* T-3′, R3/131PCV224-233 forward: 5′-GGG **GGA TCC ACC ACT AAA ACC** ***A*** *TC CGA TCT TTC CGT CGC CG*-3′, R3/131PCV224-233 reverse: 5′-GGG **GGA TCC TTA AGT TGA ATC C** *TG AAG GTA CTT TCC GAA CTG TAA CCC*-3′. (The RE recognition sequences are underlined, the inserted PCV derived regions are bold and the sequences complementary with the viral sequences are italics.) The PCR reactions were carried out with *Pfu* polymerase (Fermentas) to minimize the chance of non-intentionally introduced mutations. After the PCR reaction the product containing the full length clone terminated with the PCV derived epitope sequence was digested with the appropriate restriction enzymes (*Sn*aBI or *Bam*HI) and the plasmid was recircularized. The nucleotide sequence of the recombinant clones was confirmed by automated dideoxy sequencing. Schematic graphical illustration of the plasmid constructions were previously published by Nuzzaci et al. [19] and Vitti et al. [20].

### Plant Inoculation

Plants were inoculated at the three-leaf (*Nicotiana tabacum L.* cv. Xanthi) or six-leaf (*N. clevelandii*) stage with purified virions or with *in vitro* transcripts. For plant inoculation 20 µg/ml virion was used in 0.03 M Na_2_HPO_4_, (pH 8.7). The constructs were transcribed *in vitro* with T7 RNA polymerase (Fermentas). For inoculation, 2 µg of each of the RNA 1, 2 and 3 transcripts were used per plant in 50 µl 50 mM Na_2_HPO_4_, pH 8.6. The plants were kept in environmentally controlled growth chambers with a cycle of 14 h of light (22°C) and 10 h of dark (18°C). Chimeric viruses were tested under confinement conditions in accordance with national regulations. The average virion production rate was 70 mg per 100 g of leaf material.

### Analysis of Plants

Total RNAs were extracted from 200 mg systemically infected leaves 21 days after inoculation [38], [39]. Approximately 100 ng total RNA was denatured with formaldehyde and formamide (Sigma-Aldrich), electrophoresed in formaldehyde-containing agarose gels and blotted onto nylon membranes [40]. Northern blot hybridization analysis was performed with random-primed ^32^P-labelled DNA fragments specific for the CMV RNA3 sequence as described previously [36].

Virions were purified according to the method described by Lot et al. [41]. RNA was extracted from virions with phenol and SDS. RT-PCR analysis was carried out with the CMV 3′ end primer (5′-GGCTGCAGTGGTCTCCTTATGGAGAACCTGTGG-3′) and a forward primer located at nucleotide position 1273–1292 of RNA3 (5′-CGTCGTCGCCCGCGTAGAGG-3′). The nucleotide sequence of the amplified fragment was determined.

### Detection of PCV2 Cap Epitope by ELISA

The presence of the PCV2 epitope in the recombinant CMV particles was detected by an indirect enzyme linked immune sorbent assay (ELISA). Recombinant or wild type virus particles were released, concentrated and purified from infected plants by standard methods [41]. The presence of the epitope was tested by the use of a PCV2 specific hyperimmune polyclonal swine antiserum previously produced by a series of PCV2 vaccinations and subsequent booster infection [42]. PCV2 negative pig serum was used as negative control. The viral antigens were dissolved by 1% sodium laurylsarcosinate (Sigma-Aldrich) and diluted to a concentration of 1 mg/ml in phosphate buffered saline (PBS), plated in twofold serial dilutions, 100 µl/well, into ELISA plates. The plates were incubated overnight in a humid chamber at 4°C. Tenfold dilutions of the hyper immune serum were applied in a checkerboard fashion in a standard ELISA protocol, using horseradish peroxidase labeled anti-pig IgG conjugate (Sigma-Aldrich) to detect bound antibodies. Incubations were performed for one hour at room temperature, with 3 washes between each step, using 150 µl of washing buffer (PBS, 0.05% Tween 20) for each well. Wild type CMV served as negative antigen control.

### Immunization of Mice

Both recombinant and wild type CMV preparations were used for the immunization of mice in order to test the immunogenicity of the PCV2 epitopes. Fifteen female SPF mice (CRL: NMRI BR, Charles River, USA) of 8 weeks of age were divided into 3 groups. Group 1 received the wild type virus, group 2 a recombinant CMV carrying the PCV2_224-233aa capsid protein sequence and group 3 was mock immunized with PBS. The vaccines were applied intraperitoneally as mixtures of 100 µl (10 µg) of the antigen (or PBS) mixed with equal volume of incomplete Freund’s adjuvant (Sigma-Aldrich). Mice were vaccinated twice with a 2 weeks interval and exterminated 30 days after the first injection. Blood was collected and immune sera separated.

### PCV2 Challenge of Immunized Pigs

Thirteen conventional PCV2 free (confirmed by PCR and antibody detecting IF test) piglets were weaned at 2 weeks of age and divided into 3 groups. The experiments started at the age of 4 weeks. Five piglets (group 1) were injected intramuscularly with 2 mg of the recombinant CMV, mixed with incomplete Freund’s adjuvant, in a total volume of 2 ml. Five piglets (group 2) were vaccinated with inactivated PCV2 vaccine (Circovac, Merial). Immunizations were repeated after 10 days. The remaining 3 animals were mock immunized (group 3). Ten days after the second immunization all of the piglets were challenged with virulent live PCV2 (strain R15, kindly provided by dr. A. Cságola, Szent István University, Budapest, Hungary), administered both orally (5×10^3^ TCID_50_) and intraperitoneally (2×10^3^ TCID_50_). Piglets were examined for clinical sings of PCVDs (diarrhea, respiratory distress, skin lesions, general condition) and blood samples were collected before the first vaccination and once a week during the entire study for PCV2 antibody testing. The piglets were euthanized following general anesthesia on the 23^rd^ day after challenge [43]. Post mortem examinations were carried out; organ samples (heart, lung, liver, spleen, kidney, tonsil, mediastinal and inguinal lymph nodes) were collected from each animal and stored at −20°C until processing.

### Immune Fluorescence Test

Sera from the immunized mice and pigs were analyzed for the presence of PCV2 specific antibodies in an indirect immune fluorescence assay, according to standard protocols. Briefly, *Spodoptera frugiperda* (*Sf*) 9 insect cells in 96 well tissue culture plates were infected in parallel rows with a recombinant baculovirus producing PCV2 capsid protein and with wild type baculovirus (*Autographa californica* nuclear polyhedrosis virus), both with a multiplicity of infection of 1 per cell, or were left uninfected. The recombinant baculovirus produced with the BacPAK™ Expression System (Clontech) was kindly provided by dr. A. Cságola. Cell cultures were fixed after 2 days of incubation with 200 µl of an ethanol:acetone 1∶1 mixture for each well. After 10 minutes the fixed cells were air dried and used for the indirect immune fluorescence tests. The sera collected from mice and pigs were applied in twofold dilutions starting with 1∶5, the results were evaluated after the addition of mouse or pig specific FITC conjugated antibodies (Sigma-Aldrich) in a fluorescent microscope.

### Detection of PCV2 DNA in Infected Pigs

Samples were homogenized using TissueLyser II (Quiagen) and viral DNA was extracted using the InnuPREP Virus DNA/RNA Kit (Analytik Jena AG) according to the manufacturer’s instructions. Quantitative real-time polymerase chain reaction (qPCR) was used to detect PCV2 DNA. Primers (KCV-F: 5′- AAGTAGCGGGAGTGGTAGGA-3′ and KCV-R: 5′-GGGCTCCAGTGCTGTTATTC-3′) and the TaqMan probe (KCV-P: 5′FAM- TCCCGCCATACCATAACCCAGC-3′BHQ1) were designed for the PCV2-R15 capsid gene sequence with the online tool (https://www.genscript.com/ssl-bin/app/primer). To determine copy numbers, a serial dilution of pET6xHN plasmid (Clontech) with the PCV2 capsid gene insert was used as internal standard. The sensitivity of the test was between 10 to 100 copies/reaction. Duplicate PCR reactions were performed in 50 µl reactions, each containing 36.3 µl water, 5 µl DreamTaq buffer (Fermentas), 1 µl (0.5 mM) MgCl_2_ (Fermentas), 1 µl (0.2 mM) dNTP mixture (Fermentas), 0.5 µl of each primer and the probe (0.1 µM), 0.2 µl (1 unit) polymerase (DreamTaq, Fermentas) and 5 µl sample DNA. Reactions were performed in the Mastercycler Realplex thermocycler (Eppendorf) as follows: 94°C for 2 minutes, 30 cycles of 94°C, 30 s, 60°C 30 s and 72°C, 45 s, followed by cooling to 25°C.

## Results

### Epitope Prediction on the PCV2 CP Model

**Figure 2:**
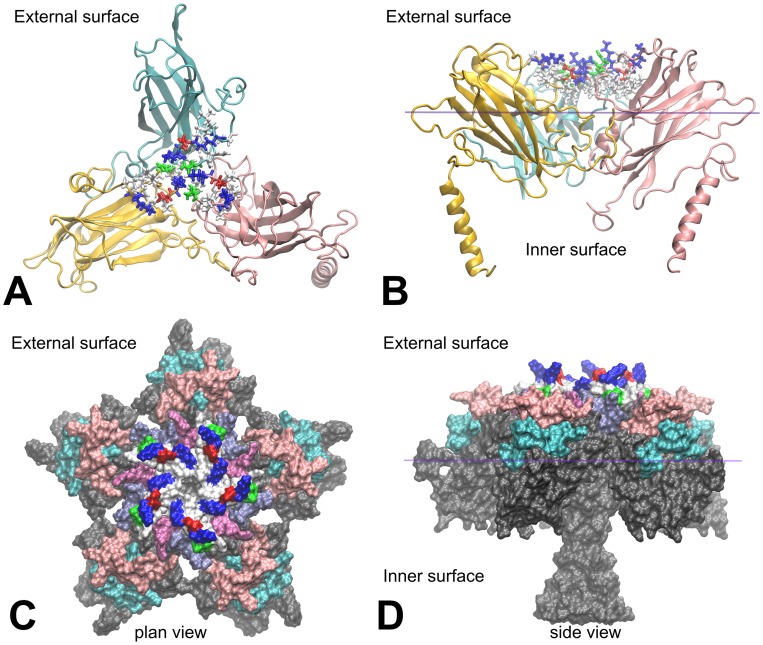
CMV and PCV epitopes on the virion surface. Spatial localization of the epitopes on the modified CMV CP trimer (A, B) and on the predicted PCV2 pentamer surface (C, D). The epitope (PCV2_224-233) was inserted after the 131 aa position. The CMV subunits A, B and C are cyan, pink and gold, respectively. The epitope is illustrated in licorice representation. The basic amino acids are blue, acidic amino acids are red, the non-polar amino acids are gray and the polar amino acids are colored in green. Plan view of the external surface of the predicted PCV2 CP pentamer (C) and the side view of the pentamer showing the outer surface and the inner surface part (D). External PCV2 capsid protein epitope colors: PCV2_37-43 is ice cube, PCV2_90-96 is mauve, PCV2_126-145 is cyan, PCV2_169-186 is pink and PCV2_224-233 is colored with the above used color schemes.

**Figure 3:**
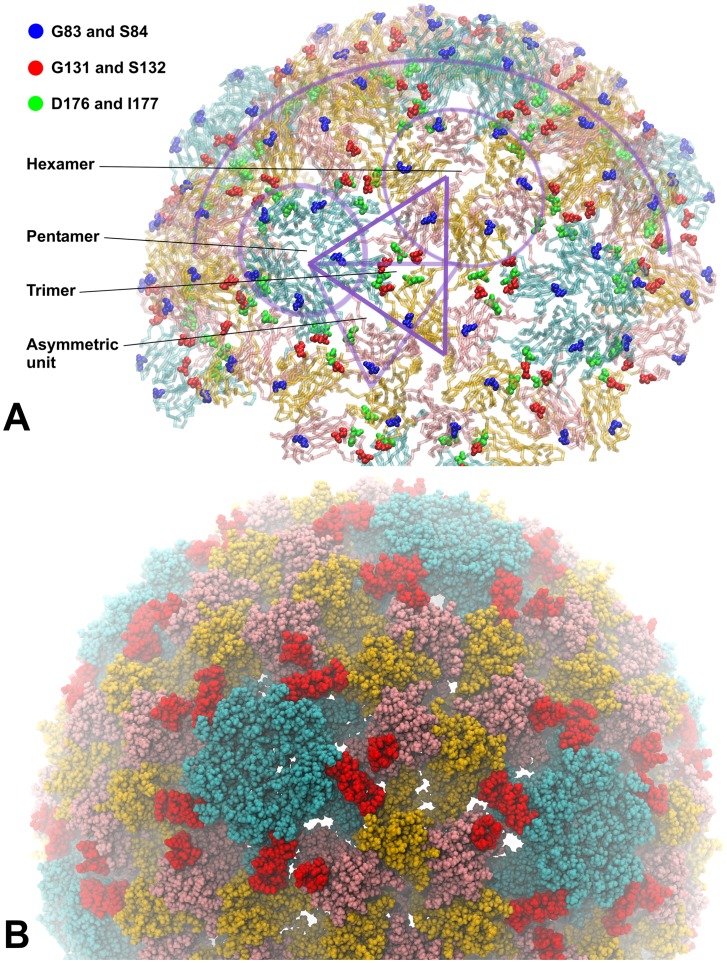
Visualization of epitope insertion points in CMV. Localization of stable epitope insertion sites G83/S84 (blue), G131/S132 (red) and D176/I177 (green) in CMV. The first two sites are on the external surface of the virion while the third is located towards the inside of the virion (A). The CMV subunits A, B and C are cyan, pink and orange, respectively. The symmetrical distribution of the inserted epitope (red) is visible on the Van der Waals representation of the modified CMV virion surface (B).

**Figure 4:**
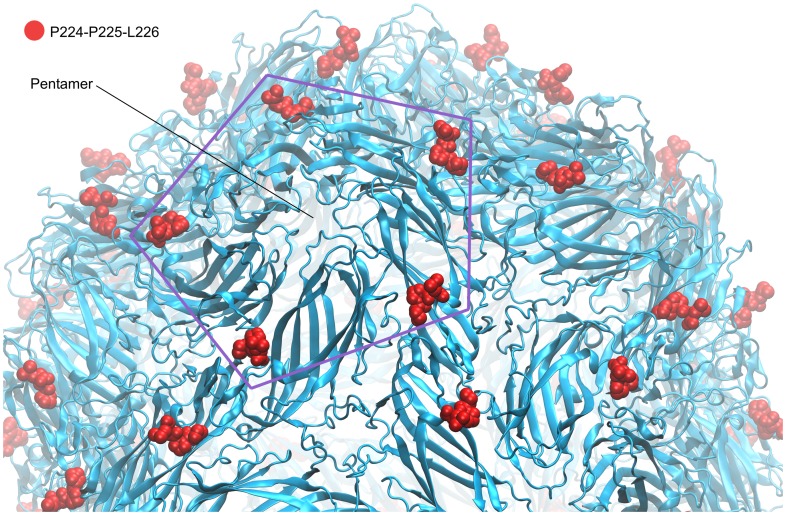
X-ray structure of the PCV2 virion (PDB ID code: 3R0R). The last seven residues are not present in the X-ray structure but it is well visible that the C-terminal tail (red) of the PCV2 capsid protein is located at the edge of the CP pentamer.

A PCV2 CP pentamer model was created from the predicted PCV2 CP so that the N-terminal α-helices form parallel bundle along a fivefold axis. This pentamer mimicked a partial external and internal surface of the PCV2 virion (Fig. 2, C and D). Five outer loop regions were identified on the basis of visual observation of the PCV2 pentamer three-dimensional model as potential epitopes during immune response. The predicted PCV2 epitope sequences, indicated by their nucleotide positions and length in amino acids (in parenthesis) were as follows: PCV2_37-43(7): RWRRKNG, PCV2_90-96(7): SIPFEYY, PCV2_126-145(20): DDNFVTKATALTYDPYVNYS, PCV2_169-186(18): STIDYFQPNNKRNQLWLR, PCV2_224-233(10): FNLKDPPLKP. On the basis of spatial distribution it was predicted that the PCV2_224-233 epitope bundle may trigger a major immune response as it is localized at the centre of the CP pentamers (Fig. 2 C and D). The 20 aa long PCV2_126-145 peptide fragment is the longest predicted epitope therefore it was selected to test its insertion properties. Insertion of the epitope to the 131aa position of CMV CP results PCV2 epitope trimer bundles in the centre of the CMV CP trimers of the virion surface (Fig. 2 A and B, Fig. 3). The X-ray structure of the PCV2 virion was published in August 2011 by Khayat et al. [44]. The experimentally determined PCV2 structure partially confirmed the relevance of our previously predicted model. In the above mentioned study it was confirmed that the C-terminal tail of the capsid protein indeed localized to the PCV2 virion surface (Fig. 4).

### Expression of PCV2 CP Derived Epitope on CMV Surface

Three stable epitope insertion points were detected in CMV to date [19], [20]. The built-in epitopes at these points did not block the propagation of CMV and the long-distance movement in plants. Two of them are displayed on the external surface of the virion while the third protrudes towards the inside of the virion (Fig. 3). There is an electrostatic limitation to express epitopes facing the inside of the virion as only the expression of epitopes with positive charge, that do not interfere with the RNA-binding of the inner surface of the CMV capsid can successfully be attempted.

**Figure 5:**
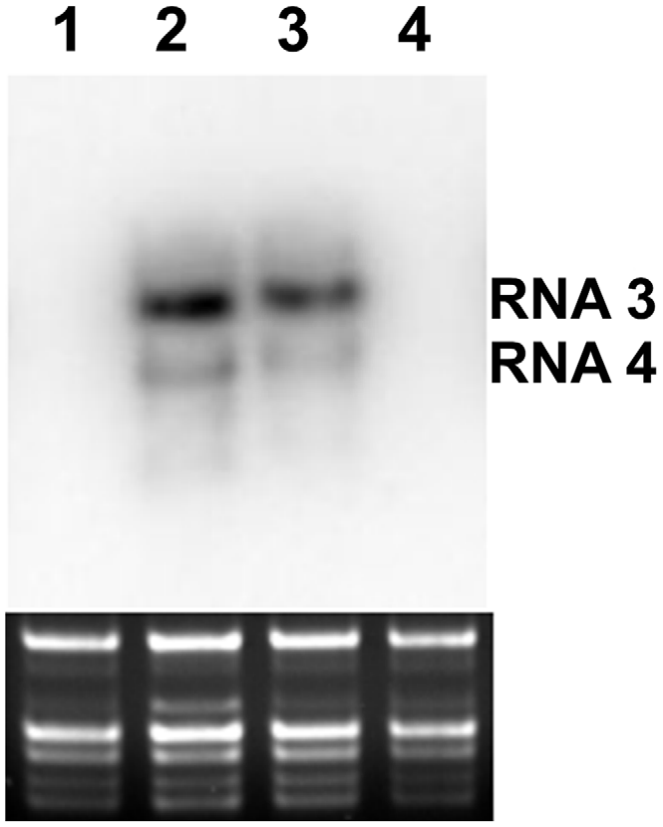
RNA detection. Northern blot analysis of accumulated viral RNAs in systemically infected leaves of *N. clevelandii* Gray plants 14 days after the inoculation. The hybridization probe was specific to RNA3 of CMV. Ethidium bromide-stained rRNA from the same volume of each sample is shown below each lane. Lane 1: mock inoculated, 2: R-CMV, 3: pR3/131-PCV224-233, 4: pR3/131-PCV126-145.

Two out of the five predicted epitopes were inserted after 131 aa position of the CMV CP. *Nicotiana clevelandii* Gray and *N. tabacum* L. cv. Xanthi plants were inoculated with the recombinant and control *in vitro* transcripts in the presence of R-CMV RNA 1 and 2 transcripts. Systemic symptoms were observed 6–8 days after the inoculation in the case of the control infection (R-CMV), after 8–10 days in the case of the pR3/131-PCV224-233 construct, but symptoms never were observed in the case of the pR3/131-PCV126-145 construct. The Northern analysis confirmed the visual observation (Fig. 5).

In the upper, not inoculated leaves the recombinant virus was detected in the case of the pR3/131-PCV224-233 construct, but not in the case of the pR3/131-PCV126-145 14 days after the inoculation. The virus was further propagated on *N. clevelandii* Gray plants and purified ten days after the inoculation. The yield of the virus purification in the case of pR3/131-PCV224-233 was similar to the purification yield of the wild type R-CMV. The purified virus RNA was analyzed by RT-PCR and nucleotide sequence determination. The integrated epitope sequence proved to be stable after one month propagation in test plants. Electron microscopic studies proved the identical size and shape of the recombinant (R3/131-PCV224-233) and the R-CMV virus particles (data not shown). Nevertheless after 5–6 months propagation and serial passages on the *Nicotiana clevelandii, Nicotiana benthamiana or Nicotiana tabacum* cv. Xanthi plants the complete deletion of the PCV2 epitope was observed. During long term maintenance in a few cases residue 131 of the CMV coat protein mutated to Pro or Ser after the insert deletion. Optimization of propagation time, appropriate plant species selection and effective growth condition development will be required to further increase the stability of the epitope.

### Antigenicity and Immunogenicity of the Recombinant PCV2 Epitopes

**Figure 6:**
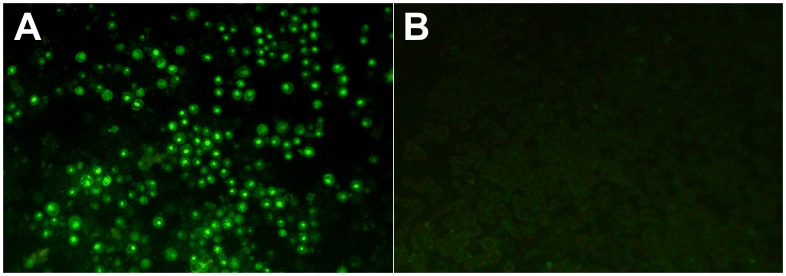
Detection of PCV2 specific antibodies. Indirect immune fluorescence test results of sera from mice immunized with recombinant (A) or wild type (B) CMV, detected in PCV2 capsid expressing baculovirus infected cells. The pictures show the results obtained with 1∶40 serum dilution of both antibodies.

**Figure 7:**
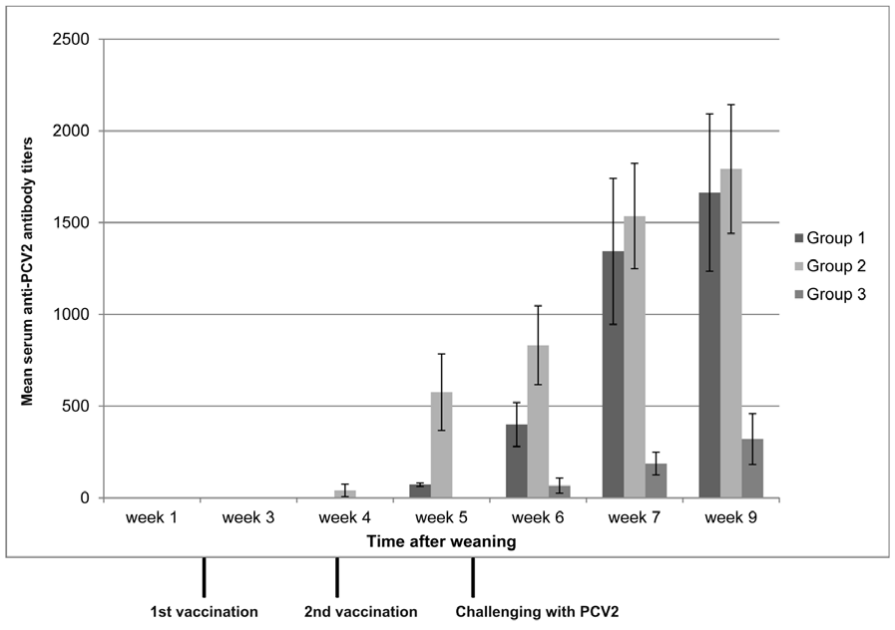
Anti-PCV2 antibody response of pigs. Bars indicate the change of the antibody titers of pigs immunized by recombinant CMV-PCV2_224-233 (group 1) or inactive PCV2 (group 2) or mock (group 3) and challenged with PCV2, as measured by indirect immune fluorescence test.

The ELISA test showed that the PCV2 epitope was present in the CMV capsid, as the polyclonal PCV2 specific pig antibodies reacted with the recombinant construct but not with the wild type virus. The titer (1∶10000) of the pig serum measured with the original PCV2 virus antigen was lower (1∶100) when using the recombinant virus. The immune sera collected from mice vaccinated with the recombinant CMV construct induced PCV2 specific antibodies, as indicated by the immune fluorescence test (Fig. 6). The titer of the antibodies was between 1∶80 and 1∶320. PCV2 specific antibodies in pigs appeared after the second immunization in group 1, after the first immunization in group 2 and only after challenge in group 3 (Fig. 7). No PCV2 DNA was detected in group 2 and the virus was demonstrated in only 2 animals after challenge in group 1. The DNA copy numbers measured by qPCR varied between 10^3^ (in lungs of one piglet) and 10^5^ (mediastinal lymph node of another piglet) in group 3. In group 1 both the lungs and the mediastinal lymph nodes of one piglet contained PCV2 DNA, but the rest of the organs were free of the virus. PCV2 was also detected in another piglet of the same group but the virus in this animal was only present in the lungs. Clinical and macroscopic post mortem examinations did not reveal any sign of clinical manifestation of the infection. The presence of subclinical disease (decreased feed conversion, effects on co-infections and vaccinations) could not be determined under these experimental conditions.

## Discussion

There is a growing need for the development of safe and efficient vaccines to prevent and control human and animal diseases, especially those of viral origin. With the development of molecular biology based technologies a number of new approaches are already being utilized in vaccine design and production. Some of them, like the baculovirus expression system have proven most efficient, but the necessity of vaccines able to induce protective immunity combined with cost effective production still remains. Plant based antigen synthesis offers the possibility of mass production of relatively inexpensive oral or parenteral vaccines. Safe production of such antigens without the risk of environmental contamination is easily provided by the use of bioreactors. Furthermore, the infectious clones utilized in this study offer a flexible, simple and rapid system for the introduction of new epitopes into recombinant particles.

Porcine circovirus was chosen as the pathogenic animal model virus for the present study. PCV infections are present worldwide and cause major losses in the pig industry. The virus is responsible for a variety of diseases (PCVDs) [45] but also it is well known for its immune suppressive nature. PCV2 infected pigs are more susceptible to secondary infections and do not respond to vaccinations as expected.

Since the successful results obtained with heterologous proteins produced in plants, either via foreign gene integration into the plant genome or expressed transiently by a replicating recombinant virus, several veterinary vaccine candidates have been produced [8] some of them are under clinical trials. Using sophisticated computer modeling methods for generating a virus capsid protein structure of the PCV2 capsid, predicted epitopes were suggested, designed and sequences representing these protein segments were inserted into the cucumber mosaic virus capsid protein. There are several experimentally described PCV2 epitopes which were mainly determined by immunological epitope mapping methods [21]–[23], [46]. Some of the predicted epitope sequences overlapped with the experimentally mapped epitopes but the lengths were not equal. This difference is due to the determination method of the predicted PCV2 epitopes because our determination has been based on three-dimensional modeling of PCV2 CP pentamer which represents a part of the PCV2 virion surface while the monomer PCV2 CP or its peptide fragments (PEPSCAN) were used in the epitope mapping experiments. Based on structure-based epitope prediction methods we were able to select a potential PCV2 epitope which can induce an efficient immune response.

Our earlier studies [31] determined several loops on the virion surface of CMV that were essential for the formation of infectious virus particles. Loops were also identified which can be changed without loss of virus stability and infectivity. During the present study the virion models were created utilizing the Oligomer Generator application with the constructed asymmetric units using A, B and C coat proteins of the plant virus. Three suitable insertion sites were determined by Nuzzaci et al. [19] and Vitti et al. [20] on the CMV CP. The insertion points are summarized in Figure 3. In terms of immunization the following conclusions can be drawn based on the conformational features of the insertion positions: 1- Insertion point 83–84 is located at the end of the βB-βC loop of the CMV CP. The built-in epitopes are expressed on the surface of CMV virions and are located far apart. Thus, production of antibodies might be induced by the assembled virion or the monomeric capsid proteins. 2- Insertion point 131–132 is located in the middle of the βE-αEF loop of the CMV CP. The above mentioned conclusion is true for this insertion point as well but this position has the advantage that the inserted epitopes form a tripartite group in the middle of the CMV CP trimers (Fig. 2 A and B). So these epitope bundles may allow the production of antibodies more efficiently. 3- Insertion point 176–177 is located in the middle of the βG-βH loop. In this case the inserted epitopes are expressed on the inner surface of CMV virions therefore this construction can only induce immune response after virion disassembly.

In our study only the pR3/131-224-233 construct was infectious on different test plants, but the pR3/131-126-145 constructs never induced systemic infection. While in the first case a 10 aa long fragment was integrated into the CMV CP, in the second case a 20 aa long region was introduced into the same position. Possibly there is a size limit of the inserted sequence tolerated by the virus. In the same position a 12 aa long Alzheimer disease derived oligopeptide was tolerated, but a 15 aa long derivative was also unstable even after one passage on *Nicotiana tabacum L.* cv. Xanthi plants [20]. Probably above a certain size, depending also on the secondary structure, the inserted peptide interferes with virus replication and particle assembly.

The presence and proper appearance of the epitope derived from PCV2 capsid on the CMV virion surface was verified by its ability to bind to PCV2 induced antibodies and also by its ability to induce such antibodies both in mice and pigs. Although the antibodies were demonstrated in pigs, they only appeared after the second dose of vaccine, indicating that either the amount of injected antigen was too low for pig immunization or that the single epitope of the PCV2 capsid is relatively less immunogenic than the entire capsid protein. According to the qPCR results, vaccination with the recombinant CMV decreased virus spread in the challenged animals, although somewhat less efficiently than the control vaccine. Effects of the vaccination on clinical or subclinical manifestations of PCVDs could not be evaluated in this experiment, because the reproduction of the disease is hard to achieve with PCV2 inoculation only [45], since PCV2-related pathomechanisms are activated by modulation of the immune system or infections by other pathogens [47]. Based on the antibody and the challenge results it was concluded that PCV2 specific vaccines can be produced by CMV expression but further experiments are needed to determine the most suitable vaccination protocol, in terms of dosage, frequency and perhaps also the adjuvant to be used.

According to these results the CMV vector proved suitable for the accurate expression of the inserted PCV2 epitope. Experiments will be needed both in mice and in pigs to determine if the given or further constructs are able to induce not only a systemic immunity following parenteral vaccination but also mucosal immunity against PCVDs when fed to the animals.
